# Circular RNA Regulation of Myogenesis

**DOI:** 10.3390/cells8080885

**Published:** 2019-08-13

**Authors:** Pengpeng Zhang, Zhe Chao, Rui Zhang, Ruoqi Ding, Yaling Wang, Wei Wu, Qiu Han, Cencen Li, Haixia Xu, Lei Wang, Yongjie Xu

**Affiliations:** 1 Department of Biotechnology, College of Life Sciences, Xinyang Normal University, Xinyang 464000, China; 2 Institute of Animal Science and Veterinary Medicine, Hainan Academy of Agricultural Sciences, Haikou 571100, China

**Keywords:** circular RNA, circRNA, skeletal muscle, myogenesis

## Abstract

Circular RNA (circRNA) is a novel class of non-coding RNA generated by pre-mRNA back splicing, which is characterized by a closed-loop structure. Although circRNAs were firstly reported decades ago, their regulatory roles have not been discovered until recently. In this review, we discussed the putative biogenesis pathways and regulatory functions of circRNAs. Recent studies showed that circRNAs are abundant in skeletal muscle tissue, and their expression levels are regulated during muscle development and aging. We, thus, characterized the expression profile of circRNAs in skeletal muscle and discussed regulatory functions and mechanism-of-action of specific circRNAs in myogenesis. The future investigation into the roles of circRNAs in both physiological and pathological conditions may provide novel insights in skeletal muscle development and provide new therapeutic strategies for muscular diseases.

## 1. Introduction

The skeletal muscle is the largest organ in animals, which constitutes 30~–50% of the body mass. Skeletal muscle plays an important role in locomotion and metabolism. Therefore, proper muscle growth and homeostasis are the critical determinants of human motor performance. Conversely, muscular diseases, such as muscular dystrophy, sarcopenia, atrophy, and cachexia, severely decline the life quality of humans [1]. Numerous experiments have established that the development and growth of skeletal muscle mainly rely on the proliferation and differentiation of myogenic stem cells. Though the origin of myogenic stem cells varied, most of them are derived from the mesodermal cell lineages [2,3]. At first, the myogenic stem cell is marked by the expression of the paired box genes, such as Pax3 and Pax7 [4]. Next, many transcription factors are involved in regulating the activity of myogenic stem cells and among which the most important regulator is called muscle regulated factors (MRF), including Myod, myf5, myogenein and Mrf4. All of the MRFs, together with Pax7 and Pax3 finely control myoblasts proliferation and differentiation [5]. At last, differentiated myoblasts fuse with each other to form multiple nuclear myotubes under the control of myomaker and myomixer [6,7].

Despite the above essential protein-encoding genes, abundant research in recent years has focused on non-coding RNAs, such as microRNA and long-non coding RNAs, which also have important regulatory roles in skeletal muscle growth and development [8]. Recent emerging evidence indicates that circRNA is another type of non-coding RNA. CircRNAs are attracting widespread interest because they play important roles in normal tissue development and disease progression. Although human circRNAs were first discovered in the 1990s, little attention has been paid to their functions. At that time, they were considered abnormal splicing products resulting from splicing errors [9]. In addition, circRNAs are often of low abundance and the traditional methods to study linear RNAs are not applicable. Until recently, with the development of biochemical enrichment methods and the progress of high throughout RNA-seq, circRNAs have been identified in a large number of species. Though most circRNAs are expressed at low levels, some of them are more abundant than their linear counterparts [10,11]. Accumulated evidence has revealed that circRNAs are evolutionally conserved and their expression levels are tissue and developmental stage-specific, indicating that circRNAs can have regulatory functions [12,13]. Interestingly, recent studies reveal that circRNAs are abundant in skeletal muscles and global expression levels of circRNAs dynamically change during myoblasts differentiation [14,15]. In addition, several circRNAs have been demonstrated with key roles in muscle development and growth. Here, we highlight recent advances in our understanding of circRNAs biogenesis and expression in skeletal muscle, with a particular focus on their functions and mechanisms in myogenesis.

## 2. CircRNA Biogenesis

Though the biogenesis of circRNAs is poorly understood, it has been suggested to be related to canonical splicing, as splicing signals are generally found to be flanking the junction regions of circRNAs. Mutation of the splicing sites resulted in low efficiency of the circulation. In addition, inhibition of the canonical spliceosome decreases both linear RNA and circRNA levels [16]. However, the expression levels of circRNAs are not always correlated with their linear counterparts, suggesting that spliceosome can discriminate linear splicing from RNA circulation, through a yet unclear mechanism.

### 2.1. Diversity of CircRNAs

CircRNA can be distinguished from other RNA species by their closed circular structure. Due to the lack of free 3′ and 5′ ends, circRNAs are less sensitive to exonucleases and more stable than linear RNAs [13]. With regards to the genome origins, circRNAs are divided into three classes: Circular intronic RNA (ciRNA) composed of only introns; exonic circRNA (ecircRNA) is derived only from exons; exon-intron circRNA (EIcircRNA) are formed by both exons and introns [17]. EcircRNAs represent more than 80% of total circRNAs and locate in the cytoplasm. Some of the ecircRNA have been reported to have regulatory functions by interacting with microRNAs or RNA binding proteins [18]. Interestingly, ciRNAs and EIcircRNAs locate in the nucleus and regulate the expression of their parental genes [19].

### 2.2. Direct Back Splicing Model

Different classes of circRNA are generated by various mechanisms. Regarding ecircRNA and EIcircRNA, two models have been proposed, the lariat model (or exon skipping model) and direct back splicing model [20]. The most distinguished difference between these two models is the order of splicing. In the direct splicing model, the back splicing comes first. By contrast, the canonical splicing comes earlier in the lariat model [21].

**Figure 1:**
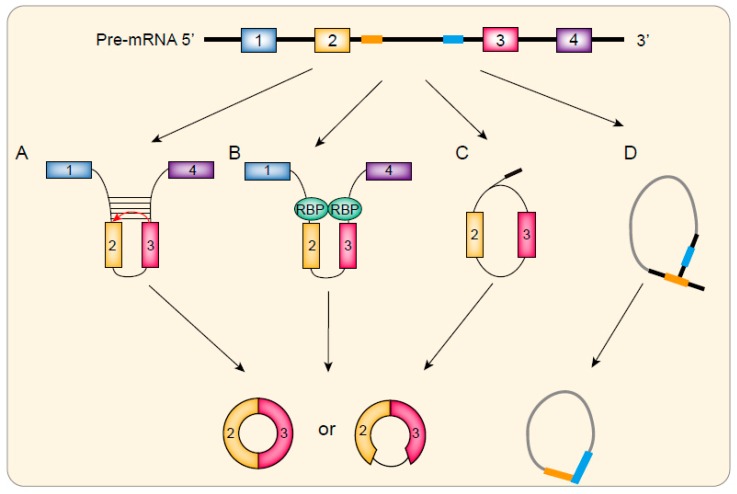
Biogenesis of circRNA. Direct back-splicing model of circRNA formation. (**A**) Reverse complementary sequence or (**B**) RNA binding proteins bring the splicing site together and facilitate back-spcling. EIcircRNA or ecircRNA are produced at last; (**C**) Exon skipping model of circRNA formation. First, the alternative produces a linear RNA and a lariat structure. Then the lariat undergoes internal back splicing and results in the generation of ecircRNA or EIcircRNA; (**D**) Model of ciRNA formation. After canonical splicing, the intron lariat is usually debrached and degraded by exonucleolytic enzyme. However, some intron lariats can escape debraching and cleaved by the exonucleolytic enzyme to form ciRNAs containing 2′- 5′ loops.

In the Direct back splicing model, the primary RNA transcript forms a stem-loop structure by flanking intron pairing or RNA-binding protein pairing [13], which brings the upstream and downstream splice signals close to each other and enables back splicing. Then, a circRNA is produced together with an exon-intron-exon intermediate, which may form a linear RNA with skipped exons or be degraded [20,22] (Figure 1A,B). This model may explain the high abundance of some circRNAs that sometimes even exceed their linear counterparts.

### 2.3. Exon Skipping Model

Exon skipping is a common pattern of alternative RNA splicing. Recent studies suggest that it also participates in circRNA biogenesis [23]. In the beginning, the alternative splicing of pre-RNA starts, as usual, then a linear RNA and a lariat structure containing the skipped exons formed. At last, this lariat undergoes internal back splicing and results in the generation of ecircRNA or EIcircRNA (Figure 1C) [20,24].

### 2.4. CiRNAs Biogenesis

CiRNAs are classified into three groups depending on the origin and biogenesis mechanisms. Both group I and group II ciRNA are rare and generated by self-splicing. Their biogenesis has been reviewed elsewhere [25]. The third type of ciRNA is common, and the biogenesis depends on spliceosome splicing. After canonical splicing, the intron lariat is quickly debrached and degraded by exonucleolytic enzyme. However, sometimes the lariat can escape debraching and inhibits the degradation [25]. It should be noted that this type of ciRNA finally forms a 2′- 5′ loop, which is different from the 3′- 5′ loop in ecircRNA and EIcircRNA. It is reported that this process relies on a consensus motif of a 7-nt GU-rich element near the 5′ spliced site and an 11-nt C-rich element near the branch point (Figure 1D) [19]. However, how does this motif act to avoid debranching is still unclear.

## 3. The Regulatory Roles of CircRNAs

**Figure 2:**
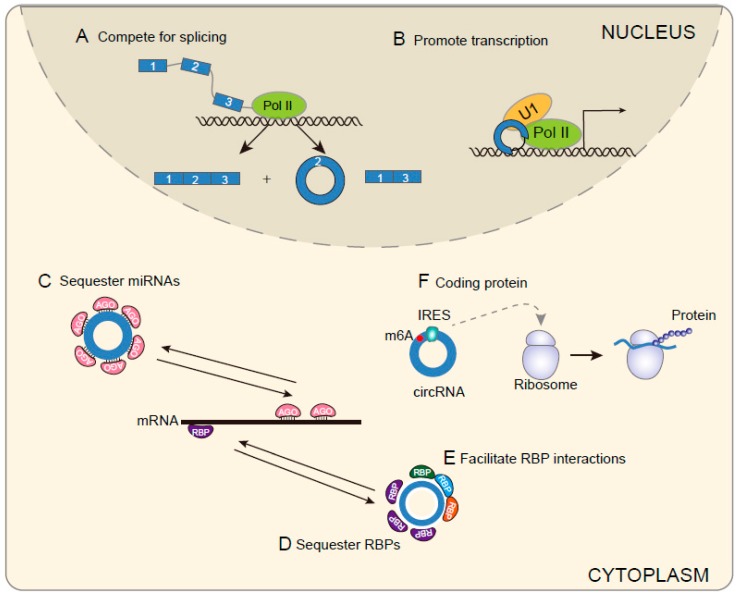
Regulatory roles of circRNAs. In the nucleus, (**A**) circRNAs can compete with their linear cognates splicing and promote exon skipping of their linear cognates. (**B**) EIcircRNAs or ciRNAs interact with Pol II, U1 snRNP at promoters of their parent genes, thus promote transcription of their parental genes. (**C**) CircRNAs serve as microRNA sponge and promote mRNA stability or protein production. (**D**) CircRNAs act as a decoy for RBP. (**E**) CircRNAs act as mediate to facilitate protein interaction. (**F**) CircRNAs encode functional protein when they harbor m6A motif or IRES.

Although the functions of circRNAs remain largely unknown, recent studies have demonstrated that circRNAs participate in many steps of gene expression, such as RNA transcription, miRNA decoy, RNA translation and protein interaction (Figure 2).

### 3.1. CircRNAs Serve as Microrna Sponge to Regulate Gene Expression

MiRNAs bind to their target mRNAs and negatively regulate mRNA stability or protein production. Recent reports have demonstrated that thousands of circRNAs harbor miRNA binding sites, indicating that circRNAs may function as competitive endogenous (ceRNAs) [26]. Some circRNAs possess multiple binding sites for a specific miRNA. For instance, CDR1as harbors more than 70 conventional miR-7 binding sites. Expression of ciRS-7 efficiently sponges miR-7, resulting in decreased miR-7 activity and enhanced levels of miR-7 targeted mRNAs [10,11]. On the other hand, some circRNAs do not possess multiple binding sites for a specific miRNA, but they harbor many different types of miRNA binding sites. For example, circITCH is a type of circRNA derived from ITCH. It is shown to sponge to miR-7, miR-17, and miR-214. All of these miRNAs can bind to the 3′-UTR of ITCH and regulate ITCH expression. Thus, circITCH acting like sponge to these miRNAs and positively regulate ITCH expression [27]. Similar to circITCH, circHIPK3 and circCCDC66 were also proved to serve as sponge to multiple miRNAs [28,29]. Taken together, these findings indicate that many circRNAs function as miRNA sponge to regulate gene expression. However, there is still debate about other circular RNAs function in the same way for some circular RNAs contain few microRNA-binding sites (Figure 2C).

### 3.2. CircRNAs Regulate Transcription

Though most of the circRNAs locate in the cytoplasm, some circRNAs are also distributed in the nucleus. It has been noted that some ciRNAs, such as those derived from introns of ANKRD52, MCM5, and SIRT7, accumulated in the nucleus [30]. Knockdown of these ciRNAs led to decreasing of their parent genes without affecting nearby parent genes, suggesting ciRNAs act in cis. A further study reported that ciRNAs accumulated at the sites of transcription. They were associated with the elongation Pol II complex and regulating transcription efficiency [30]. Similar to ciRNAs, EIcircRNAs can also accumulate in the nucleus. By performing crosslinking and immunoprecipitation with an antibody to Pol II, Li et al. identified 111 circRNAs interacting with Pol II, with all of the top 15 abundant circRNAs containing intronic sequences [31]. Furthermore, knockdown of these circRNAs led to a decrease in the mRNA levels of the parental genes and with no effect on the neighboring genes. Moreover, EIciRNAs can interact with Pol II, U1 snRNP at promoters of their parent genes. Suppression of U1 snRNA-EIciRNA interaction blocked the transcription-enhancing effect of EIciRNAs [32]. In summary, these results indicate that EIcircRNAs and ciRNAs can promote the transcription of their parental genes (Figure 2B). In addition, ecircRNA was also shown to regulate transcription. CircRNA FECR1 is derived from exons of FLI1. FECR1 was distributed in both the cytoplasm and nucleus. It bound to FLI1 promoter and positive regulated FLI1 transcription by inducing DNA hypomethylation in CpG islands of the promoter [33]. Thus, circRNAs regulate transcription not only through the transcriptional complex, but also by using epigenetic mechanisms.

### 3.3. CircRNAs Affects Splicing of Their Linear Cognates

As described above, both circRNAs and linear RNA biogenesis depend on canonical splicing machinery. Consistent with that, promotion of linear splicing efficiency led to a significantly lower number of circRNAs, indicating a negative correlation between splicing efficiency and circRNA biogenesis rates [34]. In addition, circRNAs are derived from primary RNA and contain exons of protein-coding genes; thus, circRNAs and mRNAs compete with each other for primary RNA. Accordingly, circRNA formation reduces mRNA transcript availability for protein translation [21,35]. On the other hand, circRNA can promote specific linear RNA splicing, such as in the case of circSEP3, which was produced by exon 6 of SEPALLATA3 in Arabidopsis. CircSEP3 bound strongly to its cognate DNA locus, forming an RNA:DNA hybrid, which physically slowed transcriptional elongation, and thus promoted the biogenesis of the exon-skipped alternative splicing variant [36]. Taken together, biogenesis of circRNAs can compete or promote splicing of their linear cognates (Figure 2A).

### 3.4. CircRNAs Regulate Protein Functions

CircRNAs have been shown to interact with RNA-binding proteins, increasing the possibility that they may regulate protein function by acting as protein decoy. As described above, circRNAs can bind to Pol II and U1 snRNP to regulate transcription. Moreover, the circRNA circ-Ccnb1 interacts with Ccnb1 and Cdk1 to form a ternary complex, which abolishes the roles of Ccnb1, therefore, enhancing cell proliferation and survival [37]. Similarly, the circRNA circ-FoXo3 has been reported to suppress the cell cycle process by binding to CDK2-P21 complex [38]. In summary, circRNAs can interact with RNA-binding proteins and regulate protein functions (Figure 2D,E).

### 3.5. CircRNAs Encode Proteins

Due to the lack of 5′ cap and poly (A) tails that are essential for efficient translation, circRNA was considered as noncoding RNA. However, many studies have demonstrated that circRNAs have coding capabilities [14,39,40,41]. Recent studies showed that translation of circRNAs is depended on internal ribosome entry sites (IRESs). In fact, an engineered circRNA expression vector containing IRESs could be translated to generate functional proteins [42]. CircZNF609 was one of the first endogenous circRNAs able to translate into a protein driven by IRESs and regulating myogenesis [14]. Similarly, circ-FBXW7 and circβ-catenin also encode proteins driven by IRESs. circ-FBXW7 encodes a novel protein, FBXW7-185aa, which can reduce the half-life of c-Myc by antagonizing USP28-induced c-Myc stabilization [40]. Circβ-catenin encodes a novel 370-amino acid β-catenin isoform, which uses the start codon as the linear β-catenin mRNA transcript and translation is terminated at a new stop codon. This novel protein can stabilize full-length β-catenin by antagonizing GSK3β-induced β-catenin phosphorylation and degradation [41]. Another study reported that N6-methyladenosine (m6A) motifs were enriched in circRNAs, and a single m6A site was sufficient to drive translation initiation. This type of translation was cap-independent (Figure 2F). However, it required initiation factor eIF4G2 and m6A reader [43]. These findings raise many interesting questions. For example, how does the translation is regulated, and what are the functions of these proteins?

## 4. CircRNA Expression in Skeletal Muscle

CircRNAs have been identified in numerous tissues, including brain, testis, lung, liver, heart and skeletal muscle. Numerous experiments have established that skeletal muscle is one of the tissues that are enriched in circRNAs. Many genes can generate circRNAs—for example, we previously found that approximately 36% of genes could generate circRNAs. In addition, most of the genes gave rise to more one circRNAs, while 15% of the genes generated more than 10 distinct circRNAs [15]. So far, several studies reported that the number of circRNAs in skeletal muscles or myoblast, ranges between 2000 and 37,000 [15,44,45,46,47,48]. The variation in circRNAs number might be due to the method for circRNAs enrichment, the depth of RNA sequencing, or the methods for circRNA identification (Table 1). Furthermore, circRNAs are conserved between species. Liang et al. calculated that 20.20% of pig circRNAs have human orthologs, whereas 16.96% of pig circRNAs have mouse orthologs [38]. In murine and human myoblasts, about 25% of human circRNAs were overlapped with mouse circRNAs [34].

CircRNAs have also been proved to be tissue-specific, in porcine tissue, the testis shows the largest number of tissue-specific circRNAs (1, 155), followed by the heart (205 circRNAs), muscle (174 circRNAs), and fat (147 circRNAs) [38]. These tissue-specific circRNAs are valuable regulators and worth studying further. CircRNAs have also been proved to be developmental stage-specific—for instance, 57.2–63.9% of porcine skeletal muscle circRNAs were observed at only one developmental stage [46]. Consistent with this, circRNAs expression during myoblasts differentiation progress was also noted to be dynamically changed. A further Gene Ontology analysis of the circRNA’s parental genes demonstrated that most circRNAs expressed in myoblast growth stage were related with the cell cycle, while the circRNAs expressed in the differentiation stage are enriched in development category [15]. In addition, the circRNA expression profile also altered in Duchenne muscular dystrophy and aging skeletal muscle [44].

In summary, recent studies suggest circRNAs are abundant in skeletal muscle, conserved between species and regulated in myogenesis and muscular disease.

## 5. The Functions of CircRNAs in Myogenesis

Research in recent years has established that circRNAs are key regulators of gene expression and protein functions. As previously mentioned, skeletal muscle development is a highly controlled process, which is regulated by both proteins and non-coding RNAs. Recent research suggests circRNA is the new player of the process [49,50]. Here, we summarize the current progress of circRNA in skeletal muscle growth and development (Table 2).

### 5.1. Circ-ZNF609

The human circ-ZNF609 is derived from ZNF609. Circ-ZNF609 showed higher expression in myotubes than in myoblasts and knockdown it by siRNA reduced myoblast proliferation [14]. In addition, the mouse orthologue circ-zfp-609 interacted with miR-194-5p. It is known that miR-194-5p represses BCLAF1 expression and promotes myoblasts differentiation [51]. Interestingly, circ-ZNF609 contains a 753-nucleotide open reading frame, and it is the first protein-coding circRNA identified in skeletal muscle, but so far, the function of the protein is totally unknown. Together, circ-zfp-609 inhibits myoblasts differentiation by sponging miR-194-5p and upregulation of BCLAF1.

### 5.2. CircRBFOX2

During chicken muscle development, RBFOX2 generated 11 isoforms of circRNAs, in which circRBFOX2.2-3 and circRBFOX2.2-4 were derived from exon2-3 and exon 2-4 respectively. Both of the circRNAs were expressed differentially during chicken muscle development. It was determined that circRBFOX2 contained mir-206 binding sites [48]. Previous research has proved that mir-206 involved in the cell cycle by repressing CCND2 (cyclin D2), which is an indispensable factor in cell cycle progression [52,53]. In addition, circRBFOX2 negatively regulated miR-206 expression by an unknown mechanism [48]. In summary, circRBFOX2 can sponge miR-206 and negatively regulate miR-206 expression, thus increasing CCND2 expression and promoting myoblasts proliferation.

### 5.3. CircSVIL

CircSVIL is implicated as a positive regulator of myogenesis. It is generated from exon 6 to 14 of supervillin (SVIL). The abundance of circSVIL in skeletal muscles sharply increased from E10 to E15 during chicken embryonic development and maintained at high abundance in the later stage. Four binding sites for miR-203 in circSVIL were predicted using miRanda and RNAhybrid. Further, luciferase reporter assay and Ago2 RNA immunoprecipitation showed circSVIL and miR-203 interacted with each other. It is well known that miR-203 targets c-JUN, which is an essential factor for cell proliferation [54]. miR-203 can also inhibit the expression of MEF2C, which is an important regulator of muscle development [55]. Together, miR-203 has been implicated as a negative regulator of myoblast proliferation and differentiation. CircSVIL acts as a decoy of miR-203, thus playing a positive role in myogenesis.

### 5.4. CircLMO7

CircLMO7, derived from LMO7, was highly expressed in skeletal muscle tissue. Overexpression of circLMO7 significantly decreased the expression of MyoD and myogenin (MyoG), suggesting that circLMO7 inhibited myoblast differentiation. Further analysis revealed that circLMO7 overexpression increased myoblasts proliferation and protected them from apoptosis. By performing luciferase reporter assay, it was found that circLMO7 interacted with miR-378a-3p. It is well known that miR-378a-3p inhibits HDAC4 expression [56]. HDAC4 can decrease the transcription of MEF2A and act as a repressor of myoblast differentiation [57]. In summary, circLMO7 can serve as a decoy for miR-378a-3p, results in higher expression of HDAC4 and decreases expression of MEF2A, thus promoting myoblasts differentiation.

### 5.5. CircFUT10

CircFUT10, generated by FU10, predominantly expressed in bovine skeletal muscle tissue. It showed higher expression levels in embryonic skeletal muscles than adult skeletal muscles. Overexpression of circFUT10 inhibited cell proliferation, induced myoblasts apoptosis and enhanced myoblast differentiation. RNAhybrid and TargetScan analysis revealed that circFUT10 contained three miR-133a binding sites. Further luciferase assay confirmed that circFUT10 interacted with miR-133a [58]. MiR-133a has been shown to be important in myogenesis and targets serum response factor (SRF), which is an inhibitor of myoblast proliferation [59]. In summary, circFUT10 sponges miR-133a, leading to the enhancement of SRF expression. Thus, inhibiting myoblast proliferation and promoting differentiation.

### 5.6. CircSNX29

It was noted that the expression level of circSNX29 was much higher in embryonic skeletal muscle than adult skeletal muscle. In addition, nucleoplasmic separation assay showed that it is enriched in the cytoplasm. Overexpression of circSNX29 inhibited myoblasts proliferation and facilitated differentiation. RNA hybrid showed that circSNX29 contained nine potential miR-744 binding sites. Then luciferase screening assay proved that circSNX29 directly interacted with miR-744 [60]. Next, it was found that Wnt5a and CaMKIId were the targets of miR-744. MiR-744 dramatically inhibited their expression levels and led to the activation of the non-canonical Wnt pathways, which are essential for myoblast self-renewal and muscle fibers growth [61]. Together, circSNX29 acts as a miR-744 sponge and increases Wnt5a and CaMKIId expression results in the activation of non-canonical Wnt pathways and myoblasts differentiation.

### 5.7. CircFGFR4

CircFGFR4 was highly expressed in bovine skeletal muscle. Overexpression of circFGFR4 induced cell apoptosis and promoted myoblasts differentiation. RNAhybrid and TargetScan revealed that circFGFR4 contained 18 putative miR-107 binding sites. Luciferase assay and RNA pull-down assays confirmed the interaction of miR-107 and circFGFR4. Next, wnt3a was identified as the target of miR-107 [62]. Inhibition of Wnt3a repressed myotube forming and protected myoblast from apoptosis. However, whether this is a common function of Wnt3a remains to be determined, as it has been reported that the expression of Wnt3a switched muscle stem cells from a myogenic to a fibrogenic lineage and increased connective tissue deposition [63]. In summary, circFGFR4 acts as a miR-107 sponge and increases Wnt3a expression, leading to bovine primary myoblasts differentiation.

### 5.8. CircFGFR2

CircFGFR2, generated by exon 3-6 of FGFR2, was found differentially expressed during chicken embryo skeletal muscle development [64]. Flow cytometry analysis of the cell cycle and EdU assays demonstrated that circFGFR2 accelerated myoblast proliferation. Meanwhile, circFGFR2 positively regulated myoblasts differentiation. The results of luciferase reporter assay and biotin-coupled miRNA pull-down assay suggested that circFGFR2 interacted with miR-133a-5p and miR-29b-1-5p. Further investigation discovered that miR-133a-5p and miR-29b-1-5p inhibited chicken myoblast proliferation and differentiation. Despite miR-133a-5p, miR-29 is another important myogenesis regulator, which can reduce proliferation and facilitate differentiation of myoblasts by targeting AKT [65,66]. Altogether, circFGFR2 acts as miR-133a-5p and miR-29b-1-5p sponge to promote skeletal muscle proliferation and differentiation.

### 5.9. CircHIPK3

During chicken skeletal muscle development, HIPK3 generated 11 isoforms of circRNAs [67]. CircHIPK3 was produced by the third exon of HIPK3 and differentially expressed among chicken myogenesis. CircHIPK3 promoted the proliferation and differentiation of myoblasts. In circHIPK3, three potential binding sites for miR-30a-3p were identified through miRDB and RNAhybrid analyses. Luciferase assay suggested that circHIPK3 could act as a sponge of miR-30a-3p. Further investigation discovered that MiR-30a-3p was differentially expressed during chicken skeletal muscle development and suppressed myoblasts differentiation by targeting MEF2C [55,67,68]. In summary, circHIPK3 sponges miR-30a-3p, thus increasing MEF2C expression and skeletal muscle differentiation. However, the underlying mechanism of circHIPK3 promoting myoblast proliferation needs further study.

### 5.10. CircDystrophy

The dystrophy gene is among the first genes identified to be able to generate circRNAs in skeletal muscle [69]. It is the largest human gene, consisting of 79 exons. Frame-shifting deletions or nonsense mutations of dystrophy lead to Duchenne muscular dystrophy (DMD), which is a severe muscular disease characterized by progressive muscle degeneration and weakness. In contrast to DMD, Becker muscular dystrophy (BMD) patients have milder symptoms, since they can express truncated, but partially functional protein. Mutation ranging from exon 45 to 55 of the gene represents nearly 60% of DMD/BMD cases [70]. Transforming a DMD phenotype into a BMD phenotype by 45–55 exon skipping has been proposed a new treatment strategy. Recently, Hitoshi et al. reported that eight distinct patterns of circRNAs derived from 45–55 exons and their biogenesis was related to exons skipping [71]. These results suggested that artificial and specific increase the expression levels of these circRNAs by exon skipping might have the possibility to improve or cure DMD patients. Thus, further study for the mechanism of circRNA biogenesis will be a benefit for the treatment of these muscular diseases.

## 6. Perspective

Recent studies have uncovered that circRNAs are not byproducts, but a new regulator in skeletal muscle. CircRNAs are abundant in skeletal muscle, conserved between species and regulated in myogenesis and muscular disease. Specifically, several circRNAs have been reported to regulate myoblasts proliferation and differentiation. Although the research conducted to date has greatly expanded our understanding of circRNAs, many challenging questions remain to be answered. In particular, it will be important to determine regulators involved in circRNA biogenesis. Moreover, the transportation and degradation of circRNAs in cells are challenging unanswered questions. In addition, developing high throughout methods to study circRNA functions will be of great interest, since the functions of thousands of circRNAs are still unclear.

## Figures and Tables

**Table 1:** Overview of circRNAs identified in skeletal muscle on the basis of RNA sequencing.

Organism	Sample	Treatment of RNA Library	Number of CircRNAs	Method for CircRNA Identification	References
Macaca mulatta	vastus lateralis muscle	RNase R	12,007	circExplorer	2015 [44]
Ovis aries	longissimus muscle	RNase R+	6113	-	2017 [45]
Sus scrofa	longissimus muscle	-	1489	find_circ	2017 [46]
Gallus gallus	Leg muscle	rRNA^−^, RNase R+	13,377	CIRI	2017 [48]
Bos taurus	longissimus	rRNA^−^, RNase R	12,981	-	2017 [47]
Homan sapines	Primary myoblast	rRNA^−^	2175	FindCirc	2017 [14]
Mus musculus	C2C12 cell line	rRNA^−^	1592	FindCirc	2017 [14]
Mus musculus	C2C12 cell line	rRNA^−^ and RNase R+	37,751	CIRI	2018 [15]

**Table 2:** CircRNAs involved in skeletal muscle myogenesis.

CircRNA	Biological Roles	Mechanism	References
circ-ZNF609	promotes myoblasts proliferation and inhibits myogenesis	miR-194-5p sponge	[14,51]
circRBFOX2	promotes myoblasts proliferation	mir-206 sponge	[48]
circSVIL	promotes myogenesis	miR-203 sponge	[54]
circLMO7	inhibits myoblasts differentiation and promotes myogenesis	miR-378a-3p sponge	[56]
circFUT10	inhibits myoblasts proliferation and promotes myogenesis	miR-133a sponge	[58]
circSNX29	promotes myogenesis	miR-744 sponge	[60]
circFGFR4	promotes myogenesis	miR-107 sponge	[62]
circFGFR2	promotes myoblast proliferation and myogenesis	miR-133a-5p and miR-29b-1-5p sponge	[64]
circHIPK3	promotes myoblasts proliferation and myogenesis	miR-30a-3p sponge	[67]
